# Resveratrol attenuates pyroptosis and neuroinflammation by inhibiting the TLR4/NF-κB/AIM2 pathway in ischemic stroke rats

**DOI:** 10.3389/fphar.2026.1844593

**Published:** 2026-05-15

**Authors:** Chun Liufu, Jinxuan Lin, Hui Zhang

**Affiliations:** 1 Shenzhen Maternity and Child Healthcare Hospital, Women and Children’s Medical Center, Southern Medical University, Shenzhen, Guangdong, China; 2 Hunan Provincial University Key Laboratory of the Fundamental and Clinical Research on Neurodegenerative Diseases, Changsha Medical University, Changsha, China; 3 Hunan Provincial Key Laboratory of the Research and Development of Novel Pharmaceutical Preparations, Changsha Medical University, Changsha, China

**Keywords:** ischemic stroke, neuroinflammation, neurological protection, resveratrol, TLR4/NF-κB/AIM2 pathway

## Abstract

Neuroinflammation is an important factor affecting the prognosis of ischemic brain injury and has become a potential target for stroke treatment. Resveratrol is a natural product with multiple biological activities, including anti-neuroinflammatory, anti-oxidative stress, and anti-apoptotic effects. This study focuses on the mechanism by which resveratrol protects neurological function after stroke by inhibiting neuroinflammation. A rat model of ischemic stroke was established through middle cerebral artery occlusion (MCAO). Neurological function was assessed via 2,3,5-triphenyltetrazolium chloride (TTC) staining, neurological deficit scoring, and immunohistochemical analysis. Animal behaviors were evaluated using the elevated plus maze, open field test, social interaction test, and sucrose preference test. Neuroinflammation was examined by measuring the expression of inflammatory factors including IL-6, IL-1β, and TNF-α. Oxidative stress was assessed by detecting the levels of glutathione (GSH), glutathione peroxidase (GSH-Px), superoxide dismutase (SOD), and total antioxidant capacity (T-AOC). The expression of key proteins involved in neuroinflammation- and oxidative stress-related signaling pathways was examined by Western blotting. The results demonstrated that resveratrol effectively reduced neurological deficit scores, decreased cerebral infarct volume, and alleviated post-stroke cognitive impairment (PSCI) in rats. Additionally, resveratrol significantly suppressed neuroinflammation and improved antioxidant capacity. Further mechanistic investigations revealed that resveratrol markedly inhibited the TLR4/NF-κB/AIM2 signaling pathway while activating the NRF2/NQO1 pathway. In conclusion, this study demonstrates that resveratrol exerts anti-inflammatory and antioxidant effects, and protects neurological function by targeting the TLR4/NF-κB/AIM2 and NRF2/NQO1 signaling pathways. These findings provide a novel strategy for the neuroprotective treatment of stroke.

## 1 Introduction

Stroke is a severe neurological disorder that not only causes physical dysfunction but also leads to cognitive impairment. Ischemic stroke represents the most common subtype of stroke, accounting for approximately 70% of all cases (Liang et al., 2023; Su et al., 2025; Liu et al., 2024). Thrombolytic therapy for ischemic stroke remains limited by a narrow therapeutic time window and a risk of hemorrhagic complications (Nguyen et al., 2024; Wang L. et al., 2025; Mo et al., 2025; Yao et al., 2023). In recent years, natural products have shown great potential for the treatment of neurological disorders (Gao et al., 2024; Zheng et al., 2025; Zhang F. et al., 2024; Hui et al., 2024). Resveratrol is a stilbenoid compound, mainly isolated from the roots and stems of *Polygonum cuspidatum* Sieb. et Zucc. (Polygonaceae). Accumulating evidence has verified that resveratrol exerts neuroprotective effects against various neurological disorders, including Alzheimer’s disease, Parkinson’s disease, and stroke (Tellone et al., 2015; Luo et al., 2020). Clinical studies have demonstrated that long-term administration of resveratrol can effectively improve cerebral blood flow and prevent or ameliorate vascular cognitive impairment in patients with carotid artery stenosis or occlusion (Hattori et al., 2024; Chen, Z. et al., 2025). Furthermore, resveratrol can improve blood pressure, body weight, blood glucose, and blood lipid profiles, supporting its potential use as an adjuvant agent for stroke therapy (Fodor et al., 2018; Li C. et al., 2025). Preclinical studies have revealed that resveratrol may improve neurological function after stroke via anti-neuroinflammatory and anti-oxidative stress activities (López-Morales et al., 2023). Nevertheless, the precise molecular mechanisms underlying resveratrol-mediated neuroprotection against stroke remain to be fully elucidated.

Pyroptosis, a form of programmed cell death triggered by inflammasomes, has been shown to accelerate the disruption of the blood-brain barrier and induce severe brain injury (Yuan et al., 2024; Li L. et al., 2022; Tang et al., 2025). Cerebral ischemia/reperfusion injury activates inflammasomes (such as NLRP3, AIM2), converting the precursor of caspase 1 into the cleaved form of caspase-1, causing the cleavage of GSDMD, ultimately leading to membrane rupture and the release of inflammatory cytokines (Liu P.W. et al., 2025). Additionally, activated caspase-1 converts the precursors of interleukin IL-1β and IL-18 into cleaved IL-1β and IL-18. Pyroptosis leads to further expansion of neuroinflammation after ischemic brain injury (Fu et al., 2024; Liu M. et al., 2025). Targeting upstream signaling pathways of the inflammasomes, such as the TLR4/NF-κB pathway, can effectively alleviate pyroptosis-mediated neuroinflammation and improve neurological function in stroke (Kong et al., 2021; Li M. et al., 2025; Yi et al., 2023). An increasing number of studies have demonstrated that absent in melanoma 2 (AIM2) plays a critical role in stroke-associated neuroinflammation. It is well established that resveratrol exerts neuroprotective effects by regulating the NLRP3 inflammasome and TLR4/NF-κB pathways in various brain injury models (Tufekci et al., 2021; Feng et al., 2016). However, most previous studies focused on the NLRP3 inflammasome, and the role and mechanism of resveratrol in regulating the AIM2 inflammasome in ischemic stroke remain largely unknown.

Clinical studies have demonstrated that the number of AIM2/CD68 cells is significantly increased in the penumbra of stroke patients (Matsuyama et al., 2020; Zhao et al., 2024). In both stroke animal models and oxygen-glucose deprivation/reperfusion (OGD/R) cell models, the AIM2 inflammasome is markedly activated, accompanied by neuronal pyroptosis (Zhang et al., 2025). Regulation of the AIM2 inflammasome to inhibit microglial pyroptosis can alleviate the neuroinflammatory response, mitigate ischemic brain injury, and improve neurological function (Yu et al., 2025). Another study found that AIM2 inflammasome-induced pyroptosis is a major cause of neuronal death, and AIM2 knockout significantly improved cognitive function in mice after stroke. Therefore, inhibiting the activation of the AIM2 inflammasome represents a viable therapeutic strategy for ischemic stroke (Kim et al., 2020; Lan et al., 2024; Ouyang et al., 2025). However, no safe and effective AIM2-targeting inhibitors are currently available.

In this study, we investigated the mechanism by which resveratrol inhibits pyroptosis and neuroinflammation via targeting the TLR4/NF-κB/AIM2 signaling pathway, thereby improving neurological function in ischemic stroke. These findings may provide novel targets and insights for the development of neuroprotective strategies against stroke.

## 2 Materials and methods

### 2.1 Animal experiment

**FIGURE 1:**
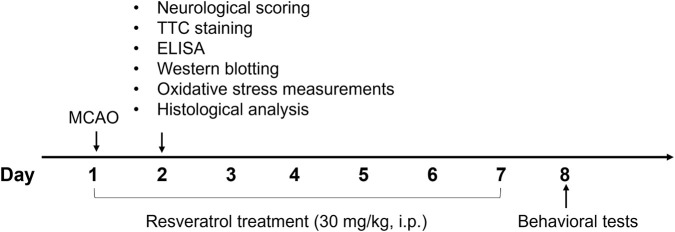
Protocol of the animal experiment. The ischemic stroke rat model was established by MCAO. Resveratrol (30 mg/kg, i.p.) was administered for 7 consecutive days. Neurological scoring, TTC staining, ELISA, Western blotting, oxidative stress measurements, and histological analysis were performed 24 h after MCAO. Behavioral tests were carried out 8 days after MCAO.

The animal experiment was approved by the Animal Care and Use Committee of Changsha Medical University. SD rats (Male, 250–280 g) were from Hunan SJA Laboratory (Changsha, China). Cerebral ischemic stroke was induced by middle cerebral artery occlusion (MCAO) (Longa et al., 1989; Zhang et al., 2023a). In brief, a suture was inserted into the middle cerebral artery of rats to occlude cerebral blood flow. After 2 h of occlusion, the suture was removed to establish cerebral reperfusion. Rats were intraperitoneally administered resveratrol at a dose of 30 mg/kg for 7 consecutive days following MCAO. Resveratrol was dissolved in a vehicle solution consisting of 5% DMSO, 10% PEG 300, 5% Tween 80, and 80% normal saline. The dose of resveratrol was selected based on a previous study (Hou et al., 2018). Rats were randomly assigned to three groups: the Sham group, the Veh + MCAO group, and the Resveratrol + MCAO group. Two separate cohorts of rats were used for this study. Cohort 1: euthanized at 24 h after MCAO, n = 9–10 per group for neurological scoring and TTC staining, n = 3–9 per group for ELISA, Western blotting, oxidative stress measurements, and histological analysis. Cohort 2: n = 11 per group, subjected to behavioral tests on day 8. The experimental protocol is illustrated in Figure 1.

### 2.2 Neurological deficit scoring

Neurological function was evaluated in each group using the Longa scoring system (Shah et al., 2006; Chen D. et al., 2025) as follows: 0 point: no neurological deficit; 1 point: forelimb weakness; 2 point: circling to the contralateral side; 3 point: inability to support weight on the affected side; 4 point: absence of spontaneous movement.

### 2.3 2,3,5-Triphenyltetrazolium chloride (TTC) staining

After rats were fully anesthetized, whole brains were harvested and stored at −20 °C. Brains were coronally sectioned into five 2-mm-thick slices. The brain slices were incubated in 1% TTC solution at 37 °C in the dark for 30 min. Following staining, slices were fixed in 4% paraformaldehyde for 30 min. Infarct volume was quantified using ImageJ software.

### 2.4 Elevated plus maze

Rats were gently placed in the central zone facing the open arm, and their exploratory behavior was recorded for 5 min. The time spent in the open arm was quantified using Smart software (Cui et al., 2024).

### 2.5 Open field test

Rats were placed in the central zone of the open field apparatus and allowed to explore freely for 5 min. The total distance traveled and the percentage of time spent in the central area were recorded using Smart software (Zhang et al., 2023b; Feng et al., 2023).

### 2.6 Social interaction test

During the adaptation phase, the test rats were placed in the central glass chamber and allowed to explore freely for 5 min, after which they were returned to their home cages. In the second phase, a familiar rat was placed in the left glass chamber, and the test rat was then placed in the central chamber and allowed to explore freely for 10 min. In the third phase, another rat was placed in the right chamber, and the test rat was allowed to explore freely for another 10 min. The interaction times between the test rat and the familiar or unfamiliar rat were recorded. The discrimination ratio was calculated using the following formula:

$$\left({\text{Time}}_{\text{Unfamiliar}\;}-{\text{Time}}_{\text{Familiar}\;}\right)/\left({\text{Time}}_{\text{Unfamiliar}\;}+{\text{Time}}_{\text{Familiar}\;}\right)\times 100\%.$$

### 2.7 Sucrose preference test

Rats were housed individually. One bottle of 2% sucrose solution and one bottle of water were provided, and the initial volumes of both solutions were recorded. To minimize positional bias, the positions of the two bottles were switched after 1 h. After a total of 2 h, the remaining volumes were recorded, and the sucrose preference percentage was calculated using the following formula:

$${\text{Sucrose}}_{\text{intake}\;}/\left({\text{Sucrose}}_{\text{intake}\;}+{\text{Water}}_{\text{intake}\;}\right)\times 100\%.$$

### 2.8 Enzyme-linked immunosorbent assay (ELISA)

The levels of inflammatory cytokines (TNF-α, IL-1β, IL-18, IL-6) in brain tissue were measured. Samples were diluted with coating buffer, added to ELISA plates, and incubated at 37 °C for 1 h. After washing three times, the primary antibody was added, followed by incubation at 37 °C for 2 h. Plates were washed another three times, and HRP-conjugated secondary antibody was added, with incubation at 37 °C for 1 h. Following a final wash, TMB substrate solution was added for color development. The reaction was stopped with 2 M sulfuric acid, and absorbance at 450 nm was detected using a microplate reader. The concentrations of inflammatory cytokines were calculated against a standard curve (Xinyu et al., 2025; Wang Q. et al., 2025).

### 2.9 GSH, GSH-PX, SOD, T-AOC

The brain tissue was homogenized using PBS and the supernatant was collected by centrifugation. The levels of GSH (Cat# A006-2-1), GSH-PX (Cat# A005-1), SOD (Cat# A001-1) and T-AOC (Cat# A015-3-1) were measured and quantified according to the manufacturer’s instructions (Jiancheng Bioengineering Institute, Nanjing, China).

### 2.10 Western blotting

Total protein was extracted from brain tissue, and protein concentration was quantified using a BCA protein assay kit. An aliquot of 20 μg protein was denatured at 100 °C and subjected to SDS-PAGE. Following electrophoresis, proteins were transferred onto a nitrocellulose membrane. After three washes with TBST buffer, the membrane was blocked with 5% skimmed milk for 1 h. The membrane was then incubated with primary antibodies overnight at 4 °C. Following three washes with TBST, the membranes were incubated with the corresponding secondary antibodies at room temperature for 70 min. Protein bands were visualized using an Odyssey CLx system (LI-COR, USA) and quantified with ImageJ software, using β-actin as the internal control. Primary antibodies were used as follows: anti-TLR4 (1:1,000; 19811-1-AP; Proteintech, USA), anti-NF-κB (1:500; ab16502; Abcam, USA), anti-p-NF-κB (1:500; ab76302; Abcam, USA), anti-iNOS (1:500; ab283655; Abcam, USA), anti-AIM2 (1:1,000; 20590-1-AP; Proteintech, USA), anti-NRF2 (1:500; D121053; Sangon Biotech, China), anti-NQO1 (1:500; D161049-0025; Sangon Biotech, China), anti-β-actin (1:5,000; AF7018; Affinity, USA). Secondary antibodies included IRDye® 680RD goat anti-mouse IgG and IRDye® 800CW goat anti-rabbit IgG (LI-COR Biosciences, USA).

### 2.11 Nissl staining

Brain tissues were serially dehydrated in 15%, 30%, and 35% sucrose solutions, then embedded in Tissue-Tek® O.C.T. compound (Sakura Finetek, USA). After freezing at −80 °C, brains were sectioned into 3-μm-thick coronal sections. Sections were immersed in Nissl staining solution (Solarbio, Beijing, China) and incubated at 37 °C for 1 h. Excess stain was removed with deionized water, and sections were differentiated in differentiation solution. The sections were then dehydrated, cleared, and mounted. Images were captured and observed under a light microscope. Three rats per group were analyzed (5 sections per rat).

### 2.12 Immunohistochemistry

Sections were incubated with 3% H_2_O_2_ at 37 °C for 10 min, then rinsed three times with PBS (5 min each). Antigen retrieval was performed by boiling in 0.01 M citrate buffer for 15 min. After cooling, sections were rinsed three times with PBS (5 min each) and blocked with goat serum working solution at 37 °C for 1 h. Sections were then incubated with primary antibodies overnight at 4 °C and rinsed with PBS, followed by incubation with biotin-conjugated secondary antibodies at 37 °C for 30 min and PBS rinsing. Horseradish peroxidase-conjugated streptavidin working solution was applied, and sections were incubated at 37 °C for 30 min before rinsing with PBS. Staining was visualized using the DAB/H_2_O_2_ chromogenic reaction, followed by rinsing with tap water. Sections were counterstained, dehydrated, cleared, and mounted. Images were observed and captured under a light microscope. Three rats per group were analyzed (5 sections per rat). The primary antibodies included anti-GFAP polyclonal antibody (1:200; IPB3211; Baijia, China) and anti-Iba-1 antibody (1:200; YP-Ab-17998; UpingBio, China).

### 2.13 Statistical analysis

All the data are presented as the means ± SEMs and were analyzed using GraphPad Prism (San Diego, CA, USA). Multiple comparisons were analyzed using one-way ANOVA with Tukey’s *post hoc* test. *p* < 0.05 was considered to indicate statistical significance.

## 3 Results

### 3.1 Resveratrol decreased neurological deficit score and reduced infarct volume in MCAO rats

**FIGURE 2:**
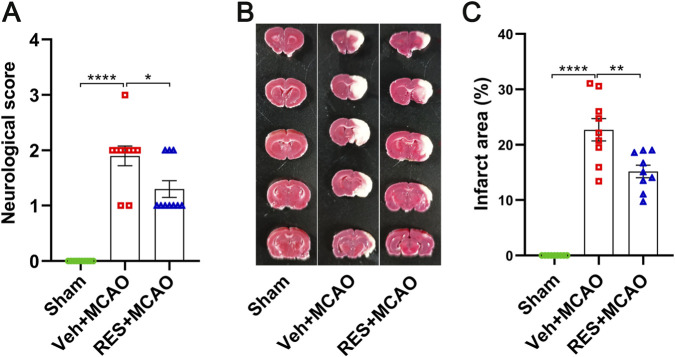
Resveratrol attenuates neurological deficits and reduces cerebral infarct volume in MCAO rats. **(A)** Neurological function scores (n = 10). **(B)** Representative TTC staining images. **(C)** Quantitative analysis of infarct volume (n = 9). The data are presented as the mean ± SEM and analyzed by one-way ANOVA followed by Tukey’s *post hoc* test. ^*^ *p <* 0.05, ^**^ *p <* 0.01, ^****^ *p <* 0.0001.

Compared with the sham group, the neurological score was increased significantly (*p* < 0.0001) in the veh + MCAO group, while resveratrol markedly reversed the increasing trend of the neurological score (*p* < 0.05) (Figure 2A). The data by TTC staining indicated that ischemia induced the significant infarction in the rats in the veh + MCAO group (*p* < 0.0001), while resveratrol significantly reduced the infarct volume (*p* < 0.01) (Figures 2B,C).

### 3.2 Resveratrol attenuated anxiety- and depression-like behaviors and improved cognitive function in MCAO rats

**FIGURE 3:**
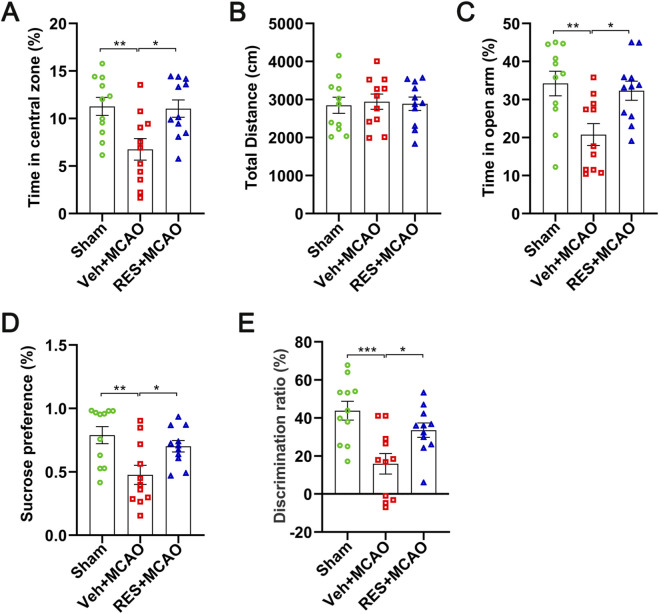
Resveratrol ameliorates anxiety-, depressive-like behaviors and cognitive impairment in MCAO rats. **(A)** The time in central zone in open field test. **(B)** Total locomotor distance in open field test. **(C)** The time spent in open arm in the elevated plus maze test. **(D)** Sucrose preference in sucrose preference test. **(E)** Discrimination ratio in the social interaction test. Resveratrol treatment significantly reversed MCAO-induced reductions in central zone time, open arm time, sucrose preference, and discrimination ratio. The data are presented as the mean ± SEM and analyzed by one-way ANOVA followed by Tukey’s *post hoc* test. ^*^ *p <* 0.05, ^**^ *p <* 0.01, ^***^ *p <* 0.001. n = 11.

According to the open-field test results, rats in the Veh + MCAO group spent significantly less time in the central area (*p* < 0.01) compared with the sham group, whereas resveratrol treatment significantly increased this time (*p* < 0.05) (Figure 3A). In addition, no significant difference in total distance traveled was observed among the three groups, suggesting that locomotor activity did not contribute to the behavioral differences between groups (Figure 3B). Consistently, data from the elevated plus maze demonstrated that rats in the Veh + MCAO group spent markedly less time in the open arms (*p* < 0.01) relative to the sham group, and resveratrol significantly restored this parameter (*p* < 0.05) (Figure 3C). Moreover, the sucrose preference test revealed that sucrose preference was significantly reduced in the Veh + MCAO group relative to the sham group (*p* < 0.01), and this deficit was significantly ameliorated by resveratrol (*p* < 0.05) (Figure 3D). Finally, the social interaction test showed that the discrimination ratio was significantly lower in the Veh + MCAO group than in the sham group (*p* < 0.001), and resveratrol significantly elevated this ratio (*p* < 0.05) (Figure 3E).

### 3.3 Resveratrol attenuated neuroinflammation and inhibiting TLR4/NF-κB/AIM2 pathway

**FIGURE 4:**
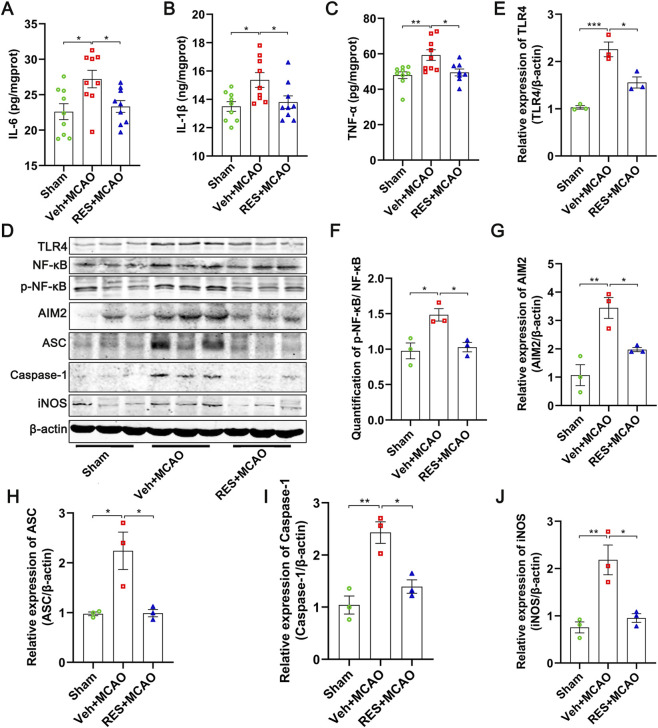
Resveratrol inhibited neuroinflammation by targeting TLR4/NF-κB/AIM2 pathway. **(A–C)** Levels of inflammatory cytokines (IL-6, IL-1β, TNF-α) in brain tissue, measured by ELISA. MCAO significantly increased cytokine levels, which were significantly reduced by resveratrol treatment (n = 9). **(D)** Representative images of Western blotting. **(E–J)** Quantitative analysis of protein expression (n = 3). MCAO markedly upregulated the expression of TLR4, p-NF-κB/NF-κB, AIM2, ASC, Caspase-1, and iNOS, while resveratrol treatment significantly reversed these elevations. The data are presented as the mean ± SEM and analyzed by one-way ANOVA followed by Tukey’s *post hoc* test. ^*^ *p <* 0.05, ^**^ *p <* 0.01, ^***^ *p <* 0.001.

The expression levels of the inflammatory factors, including IL-6 (*p* < 0.05), IL-1β (*p* < 0.05), and TNF-α (*p* < 0.01), were significantly increased in the rats in the veh + MCAO group, and were significantly reduced by resveratrol (*p* < 0.05) (Figures 4A–C). In the meanwhile, the expression levels of TLR4 (*p* < 0.001), p-NF-κB/NF-κB (*p* < 0.05), AIM2 (*p* < 0.01), ASC (*p* < 0.05), Caspase-1 (*p* < 0.01), and iNOS (*p* < 0.01), and were significantly upregulated in the rats in the Veh + MCAO group, compared with those in the sham group. Resveratrol significantly revered the expression trend of these proteins (*p* < 0.05) (Figures 4D–J).

### 3.4 Resveratrol enhanced the anti-oxidative stress ability and activating NRF2/NQO1 pathway

**FIGURE 5:**
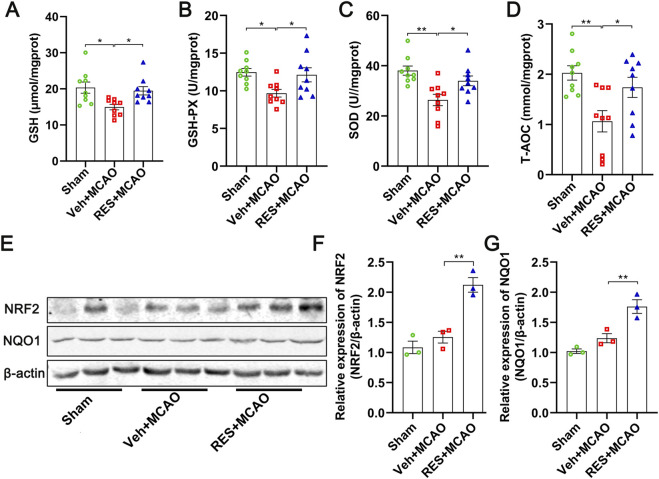
Resveratrol enhances antioxidant defense by activating the Nrf2/NQO1 pathway. **(A–D)** Levels of antioxidant indicators in brain tissue, including GSH, GSH-PX, SOD, T-AOC (n = 9). MCAO significantly reduced these antioxidant levels, which were significantly restored by resveratrol treatment. **(E)** Representative images of Western blotting. **(F,G)** Quantitative analysis of protein expression (n = 3). Resveratrol treatment markedly upregulated NRF2 and NQO1 expression, indicative of the activation of the NRF2 antioxidant pathway. The data are presented as the mean ± SEM and analyzed by one-way ANOVA followed by Tukey’s *post hoc* test. ^*^ *p <* 0.05, ^**^ *p <* 0.01.

The expression levels of the anti-oxidative stress factors, including GSH (*p* < 0.05), GSH-PX (*p* < 0.05), SOD (*p* < 0.01), T-AOC (*p* < 0.01), were significantly decreased in the rats in the veh + MCAO group, and were significantly increased by resveratrol (*p* < 0.05) (Figures 5A–D). In the meanwhile, the expression levels of NRF2 (*p* < 0.01), and NQO1 (*p* < 0.01) were significantly upregulated in the rats in the RES + MCAO group, compared with those in the Veh + MCAO group (Figures 5E–G).

### 3.5 Resveratrol ameliorates neuronal damage and inhibits inflammatory activation of astrocytes and microglia in MCAO rats

**FIGURE 6:**
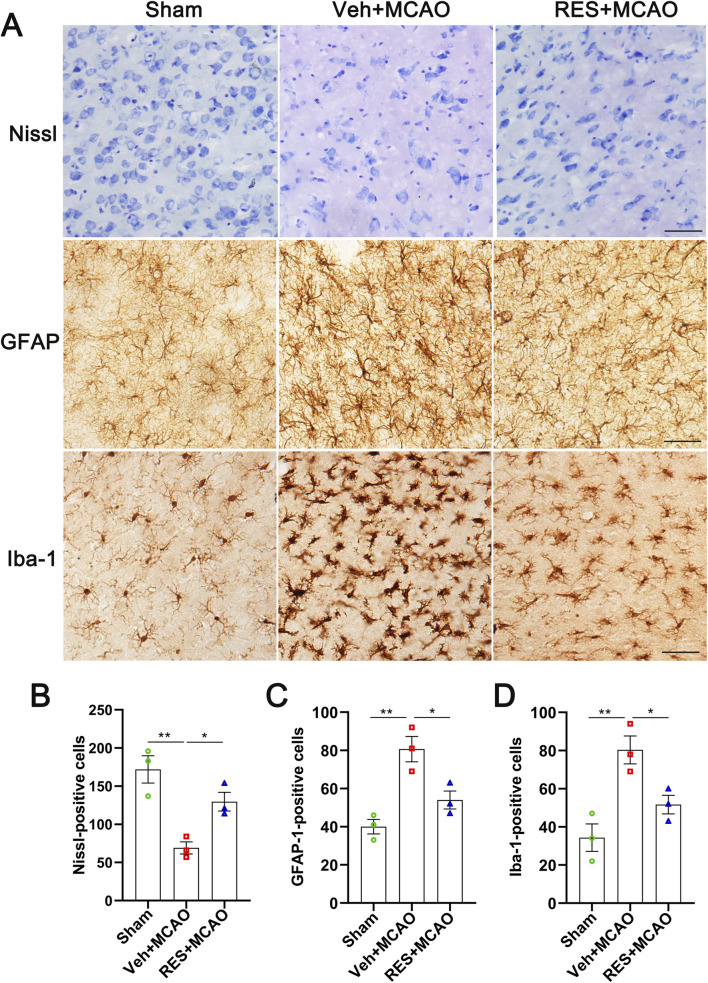
Resveratrol ameliorated neuronal loss and inhibited the over activation of astrocytes and microglia in MCAO rats. **(A)** Representative images of Nissl staining, GFAP immunohistochemistry, and Iba-1 immunohistochemistry. **(B–D)** Quantitative analysis of Nissl-positive neurons, GFAP-positive astrocytes, and Iba-1-positive microglia. MCAO caused significant neuronal loss, accompanied by robust astrogliosis and microglial activation. Resveratrol treatment significantly reversed these changes, increasing Nissl-positive cells and reducing GFAP-positive cells and Iba-1-positive cells. The data are presented as the mean ± SEM and analyzed by one-way ANOVA followed by Tukey’s *post hoc* test. ^*^ *p <* 0.05, ^**^ *p <* 0.01. n = 3.

Compared with the sham group, the volume and number of Nissl-stained cells were markedly reduced in the Veh + MCAO group (*p* < 0.01). Following resveratrol treatment, the morphology of Nissl-stained cells was significantly preserved, and their number was markedly increased (*p* < 0.05) (Figures 6A,B). Furthermore, the number and morphology of astrocytes and microglia were assessed by immunohistochemistry. Relative to the sham group, the number of astrocytes was significantly increased in the Veh + MCAO group (*p* < 0.01), accompanied by enlarged cell bodies and hypertrophic cell processes. Resveratrol treatment significantly ameliorated these morphological alterations and reduced astrocyte number (*p* < 0.05) (Figures 6A,C). Similarly, the number of microglia was also significantly elevated in the Veh + MCAO group compared with the sham group (*p* < 0.01), along with enlarged cell bodies, hypertrophic and proliferative processes, and network formation. Resveratrol significantly decreased microglial number (*p* < 0.05) and markedly restored their morphological features (Figures 6A,D).

## 4 Discussion

Neuroinflammation, as a key link in the pathological process of stroke, not only participates in the expansion of post-stroke injury but also plays an important role in neural repair and tissue remodeling (Wang et al., 2023). Pyroptosis of cells is an important pathological feature that occurs during cerebral ischemia and can lead to further expansion of neuroinflammation after ischemic brain injury, providing a promising target for neuroprotective treatment of stroke (Zhang H. et al., 2024; Hu et al., 2023; Chen Y. et al., 2025). The protective effect of resveratrol on neurological diseases has been confirmed, but the specific mechanism remains to be clarified (Dou et al., 2019; Chen et al., 2019; Liu et al., 2019). This study aims to explore the mechanism by which resveratrol inhibits pyroptosis-mediated neuroinflammation and protects the neurological function after stroke.

In this study, MCAO significantly induced neurological dysfunction and cerebral infarction in rats, confirming that rat model of stroke was successfully established (Sommer, 2017). The dose of resveratrol (30 mg/kg) was chosen based on previous studies, and lower doses were not used because they failed to produce satisfactory neuroprotective effects in ischemic stroke rats (Hou et al., 2018; Xu et al., 2024). Based on the dose conversion relationship between humans and rats, a dose of 30 mg/kg in rats is approximately equivalent to 2.4 mg/kg in humans, which is within the clinically safe and feasible range. Resveratrol effectively alleviated the neurological impairment induced by MCAO. Further, through TTC staining, it was found that after resveratrol treatment, the cerebral infarction volume in MCAO rats significantly decreased, suggesting that resveratrol can reduce the brain tissue damage in the ischemic area and alleviate the severity of cerebral infarction. This is consistent with the improvement in the neurological function score results, indicating that resveratrol has a significant protective effect on MCAO-induced cerebral ischemic injury. These results are in consistent with previous studies (Zhang et al., 2022).

The effects of resveratrol on the anxiety, depression-like behaviors and social cognitive functions of MCAO rats were further investigated. The open field test and the elevated plus maze test indicated that resveratrol significantly alleviated the anxiety-like behaviors of MCAO rats. Moreover, there was no significant difference in the total movement distance among the three groups, ruling out the interference of motor ability differences on the behavioral results. The sucrose preference test showed that MCAO induced significant anhedonia in rats, a core feature of depressive-like behaviors (Liu et al., 2025). These findings suggested that cerebral ischemic injury not only impairs neurological function but also disrupts emotional regulation, thereby contributing to the development of depressive-like phenotypes (Chen et al., 2024; Tang et al., 2023).

Resveratrol intervention effectively improves the anhedonia induced by MCAO and alleviates the depressive-like behaviors in rats. In addition to physical disabilities, stroke patients often experience poststroke cognitive impairment (PSCI), with a prevalence ranging from 20% to 80% (Sun et al., 2014). The data from social interaction experiments showed that MCAO caused social cognitive dysfunction in rats, manifested as a decline in the rats’ ability to distinguish between familiar and unfamiliar individuals. After treatment with resveratrol, the discrimination rate significantly increased, indicating that resveratrol can improve the social cognitive function of MCAO rats. This may be related to the effects of resveratrol in reducing the volume of cerebral infarction and alleviating brain tissue damage.

This study further elucidates the molecular mechanism by which resveratrol improves brain ischemic injury at the level of inflammatory response. The results showed that MCAO triggered a strong inflammatory response in the brain, and the excessive activation of this inflammatory response will further exacerbate the disruption of the blood-brain barrier, neuronal apoptosis, and cerebral infarction, which is a key link in aggravating the secondary damage after cerebral ischemia (Anrather et al., 2016; Tang et al., 2021). After the treatment with resveratrol, the increases of the inflammatory factors were significantly inhibited, indicating that resveratrol effectively alleviated the neuroinflammation after cerebral ischemia and thereby exert neuroprotective effects.

As an important pattern recognition receptor for recognizing damage-associated molecular patterns, TLR4 is activated after cerebral ischemia and initiates the downstream NF-κB signaling pathway, promoting the phosphorylation and nuclear translocation of NF-κB, thereby upregulating the transcription and expression of various pro-inflammatory factors and inflammatory effector molecules (such as iNOS), and amplifying the neuroinflammatory cascade reaction (Luo et al., 2025; Zhang et al., 2026). Meanwhile, as a key component of the inflammasome, AIM2 can further promote the maturation and release of inflammatory factors in response to ischemic injury, thereby exacerbating neuroinflammation and cell damage. The present study found that after treatment with resveratrol, the expressions of TLR4, phosphorylated NF-κB, iNOS, and AIM2 were significantly reversed. Mechanistically, resveratrol may directly interfere with the TLR4 to block downstream NF-κB activation, thereby reducing neuroinflammation and AIM2 inflammasome-mediated pyroptosis.

Furthermore, we also explored the mechanism by which resveratrol protects the neurological function of stroke through anti-oxidative stress. The results revealed that resveratrol can regulates the antioxidant stress and NRF2/NQO1 pathway, in consistence with previous studies (Farkhondeh et al., 2020). The imbalance of oxidative stress is the key factor for secondary brain injury after MCAO (Zeng et al., 2023). In our study, the expressions of GSH, GSH-PX, SOD and T-AOC in the brain tissues of the MCAO group were significantly decreased, indicating that MCAO successfully induced oxidative stress disorder, and the endogenous antioxidant system was damaged, unable to eliminate excessive reactive oxygen species (ROS), thereby exacerbating brain tissue damage. Resveratrol significantly increased the above antioxidant factors, suggesting that resveratrol can activate the endogenous antioxidant defense system and enhance the ability to clear ROS, thereby alleviating oxidative stress-induced toxic damage. Furthermore, resveratrol significantly upregulated the protein expression of NRF2 and NQO1. As the key regulator of the cellular antioxidant response, NRF2 plays a critical role in transcriptionally activating a series of cytoprotective genes, including NQO1, which neutralize oxidative stress and maintain redox homeostasis (Sun et al., 2023; Chen et al., 2024; Chen et al., 2023). Taken together, these observations suggest that resveratrol attenuates oxidative stress injury induced by cerebral ischemia, at least in part, by activating the NRF2/NQO1 signaling pathway. It is hypothesized that resveratrol activates Nrf2 signaling by disrupting the Keap1-Nrf2 interaction, promoting Nrf2 nuclear translocation and subsequent upregulation of antioxidant genes including NQO1. However, the in-depth mechanism is still to be elucidated.

The ischemia and hypoxia lead to the degeneration, damage and apoptosis of neurons, and the structural integrity of neurons is disrupted. This is an important cellular basis for the neurological functional impairment after ischemic stroke (He et al., 2021). The Nissl staining results showed that resveratrol significantly restored the morphology of neurons and increased their number. This indicates that resveratrol can effectively inhibit neuronal damage induced by MCAO and maintain the structural and functional integrity of neurons. Overactivated glial cells release a large amount of pro-inflammatory factors, ROS and other toxic substances, which aggravate neuronal damage and neuroinflammation (Chan et al., 2025; Li C. et al., 2022; Wang et al., 2022). Immunohistochemical results showed that resveratrol intervention could significantly improve the abnormal morphology of astrocytes and microglia, and reduce their numbers, indicating that it can inhibit the excessive activation of glial cells and alleviate the secondary damage mediated by them.

In conclusion, the data indicate that resveratrol exerts anti-neuroinflammatory and antioxidant stress effects by targeting signaling pathways such as TLR4/NF-κB/AIM2 and NRF2/NQO1, thereby protecting the neurological function of stroke rats and alleviating PSCI. The major novelty of our study lies in the identification of the TLR4/NF-κB/AIM2 axis as a key target of resveratrol against pyroptosis and neuroinflammation in the verification of the coordinated regulation of resveratrol on TLR4/NF-κB/AIM2 and NRF2/NQO1 dual pathways in ischemic stroke. However, it should be noted that the study still has several limitations. Firstly, we only focused on the expression changes of the TLR4/NF-κB/AIM2 signaling pathway and related pro-inflammatory factors, and did not deeply explore the upstream molecular targets of resveratrol in regulating this pathway, such as whether it indirectly acts on the TLR4 pathway by regulating molecules like SIRT1 and HMGB1, and the specific upstream mechanism of its regulation of this pathway (Karimy et al., 2020). Second, this study only used the single animal model of MCAO in rats to verify the anti-inflammatory and neuroprotective effects of resveratrol, and did not conduct verification in different species of animals or other cerebral ischemia models. Third, this study did not set up intervention groups with different doses of resveratrol, and could not clearly determine the dose-effect relationship of resveratrol’s anti-inflammatory effect, nor could it determine its optimal intervention dose and potential dose-dependent toxicity. Fourth, this study only detected the relevant indicators at specific experimental time points, did not conduct long-term follow-up observations, and could not clearly determine the long-term effects of resveratrol on neuroinflammation and neurofunctional recovery, nor could it assess its long-term safety. Fifth, the specific regulatory effects of resveratrol on different cell types (such as neurons, microglia, and astrocytes) remain to be further clarified. Finally, a sham + resveratrol group was not included in the present study. Although our findings clearly demonstrate that resveratrol exerts neuroprotective effects in MCAO rats, the addition of a sham group treated with resveratrol alone would further confirm the specificity of its action.

## 5 Conclusion

Resveratrol significantly inhibited the excessive activation of the TLR4/NF-κB/AIM2 signaling pathway, reduced the expression levels of inflammatory factors, alleviated the neuroinflammatory response in the brain of MCAO rats, and thereby improved neurological function and emotional cognitive impairment. This study provides experimental evidence for the prevention and treatment of ischemic stroke with resveratrol, and also lays a foundation for further exploration of its mechanism of action and promotion of clinical application.

## Data Availability

The original contributions presented in the study are included in the article/supplementary material, further inquiries can be directed to the corresponding author.
